# Supplementary material for: Soil microbial community responses to short-term nitrogen addition in China’s Horqin Sandy Land
Source: PLoS One. 2021 May 20;16(5):e0242643. doi: 10.1371/journal.pone.0242643 (PMC8143696; doi:10.1371/journal.pone.0242643)
Supplement: S1 Table — Sample names include the site type (G, sandy grassland; S, semi-fixed sandy land) and nitrogen addition treatment: control (CK), no N addition; N5, 5 g N m−2 yr−1; N10, 10 g N m−2 yr−1; N15, 15 g N m−2 yr−1. (DOCX) [file pone.0242643.s002.docx]

**Table S1 Phylotype coverage and diversity estimation based on the bacterial 16S rRNA gene libraries for the samples from the MiSeq sequencing analysis. Sample names include the site type (G, sandy grassland; S, semi-fixed sandy land) and nitrogen addition treatment: control (CK), no N addition; N5, 5 g N m^−2^ yr^−1^; N10, 10 g N m^−2^ yr^−1^; N15, 15 g N m^−2^ yr^−1^.**

| Samples | Valid sequences | Average length (bp) | OTUs^a^ | ACE^a^ | Chao^a^ | Shannon^a^ | Simpson^a^ | Coverage |
| --- | --- | --- | --- | --- | --- | --- | --- | --- |
| G1CK | 49599 | 414.929 | 2071 | 5006.982634 | 5020.66843 | 6.638 | 0.0051 | 0.977 |
| G3CK | 48922 | 415.525 | 2072 | 5029.104279 | 5039.553366 | 6.840 | 0.0030 | 0.978 |
| G5CK | 48342 | 414.913 | 1993 | 5100.201005 | 5016.357616 | 6.634 | 0.0038 | 0.976 |
| G1N5 | 44464 | 414.194 | 1908 | 4621.70525 | 4545.724739 | 6.593 | 0.0044 | 0.977 |
| G3N5 | 46174 | 415.509 | 2071 | 5284.121255 | 5318.214408 | 6.790 | 0.0031 | 0.974 |
| G5N5 | 48299 | 416.490 | 2051 | 5335.180163 | 5280.869634 | 6.767 | 0.0034 | 0.975 |
| G1N10 | 49870 | 412.779 | 1985 | 4819.519293 | 4763.367089 | 6.355 | 0.0072 | 0.978 |
| G3N10 | 50204 | 415.404 | 2048 | 5203.61489 | 5252.554795 | 6.722 | 0.0040 | 0.977 |
| G5N10 | 48753 | 414.907 | 2078 | 5532.818269 | 5489.310345 | 6.705 | 0.0037 | 0.973 |
| G1N15 | 45325 | 414.034 | 2038 | 5000.401802 | 4875.668863 | 6.439 | 0.0079 | 0.974 |
| G3N15 | 50234 | 415.505 | 2073 | 5313.72578 | 5291.745192 | 6.797 | 0.0032 | 0.976 |
| G5N15 | 45374 | 415.676 | 2086 | 5024.837669 | 4918.815166 | 6.781 | 0.0035 | 0.975 |
| S1CK | 40037 | 414.934 | 1811 | 4813.181102 | 4119.894737 | 6.159 | 0.0080 | 0.975 |
| S3CK | 50269 | 417.016 | 2083 | 5167.847315 | 5132.440895 | 6.817 | 0.0031 | 0.977 |
| S5CK | 46256 | 417.240 | 2012 | 4811.951076 | 4764.482206 | 6.263 | 0.0153 | 0.975 |
| S1N5 | 42645 | 414.773 | 1967 | 4748.101918 | 4821.633528 | 6.590 | 0.0045 | 0.974 |
| S3N5 | 47532 | 414.289 | 2031 | 4765.336265 | 4792.123574 | 6.559 | 0.0042 | 0.977 |
| S5N5 | 42724 | 415.112 | 2023 | 4882.953655 | 4835.371377 | 6.552 | 0.0046 | 0.973 |
| S1N10 | 45293 | 415.830 | 1993 | 4782.583382 | 4707.001739 | 6.531 | 0.0074 | 0.976 |
| S3N10 | 46721 | 416.992 | 2006 | 4842.994373 | 4825.246454 | 6.630 | 0.0045 | 0.977 |
| S5N10 | 49408 | 417.805 | 2001 | 4926.039536 | 4817.07619 | 6.433 | 0.0090 | 0.978 |
| S1N15 | 47761 | 415.475 | 1956 | 4242.927149 | 4260.24 | 6.188 | 0.0116 | 0.980 |
| S3N15 | 45381 | 416.219 | 1942 | 4386.077578 | 4284.435154 | 6.378 | 0.0071 | 0.978 |
| S5N15 | 51789 | 414.069 | 1955 | 4324.611553 | 4281.672414 | 6.194 | 0.0145 | 0.982 |

**^a^** The operational taxonomic units (OTUs) were defined using a 97% sequence similarity threshold. Abbreviations: ACE and Chao, richness estimators; Shannon and Simpson, diversity indices.
