# Supplementary material for: Soil microbial community responses to short-term nitrogen addition in China’s Horqin Sandy Land
Source: PLoS One. 2021 May 20;16(5):e0242643. doi: 10.1371/journal.pone.0242643 (PMC8143696; doi:10.1371/journal.pone.0242643)
Supplement: Attachment — Submitted filename: Reviewer Comments_PONE-D-20-34907.pdf [file pone.0242643.s005.pdf]

**Editor PLOS ONE,**

Dear Editor,

The manuscript ‘Soil microbial community responses to short-term nitrogen addition in China’s Horqin Sandy Land (PONE-D-20-34907)’ has been reviewed.

The study investigated the effect of short-term N addition on various soil microflora characteristics at three N addition levels in the sandy grassland and semi-fixed sandy land.

Overall, idea of the work is good however there are few key questions to be addressed before getting the manuscript suitable for publication in PLOS ONE. The comments are as under.

Authors need to build the rational differentiating the variable response of microbial communities upon short term and long-term nitrogen addition – it wasn’t accordingly discussed. Key words should be precise and most relevant, for example site or region presence in key words may not be important. Line 44 cut ‘they’. Authors have mentioned about the response of microbes across forest and farmland as previously reported, assumption behind studying the short-term N addition in sandy grassland and semi-fixed sandy land ecosystems is to be mentioned.

The activity of β- 1,4-glucosidasewas increased upon nitrogen addition (N5, N10), could this only be related to the release of nitrogen from plant litter degradation. Why these two particular sites with distance of 1.5 km were chosen when the pedoclimatic condition relying on were (supposedly) the same. For fungal amplification, 18S rRNA could be targeted? Did authors check for inhibition test to remove the background noise before running the qPCR assays? The bacterial gene abundances quantified through 16S RNA gene copy numbers in the sandy grassland and semi-fixed sandy showed a high variation but were there no significant differences. The microbial community structure through targeting ITS region could be a possible way to know the differences in community structure across variable soils as discussed in taxonomic composition. Community composition analyses in nicely described but dominant phyla may be described in result section. Figures could not be accessed as these were missing in the manuscript file so editor may verify the figures quality. I evaluated the work based on the detail of the manuscript provided. The conclusions should not be repetition of results rather focus the key findings.

**Sincerely,**
