# Supplementary material for: Soil microbial community responses to short-term nitrogen addition in China’s Horqin Sandy Land
Source: PLoS One. 2021 May 20;16(5):e0242643. doi: 10.1371/journal.pone.0242643 (PMC8143696; doi:10.1371/journal.pone.0242643)
Supplement: Attachment — Submitted filename: Response to Reviewers.docx [file pone.0242643.s006.docx]

25 January 2021

Reviewer

*PLOS ONE*

RE: Submission of the revised manuscript (No. **PONE-D-20-34907**): Soil microbial community responses to short-term nitrogen addition in China’s Horqin Sandy Land.

Dear Reviewer:

Thank you very much for your assistance in the review of our manuscript. We have revised the manuscript carefully according to your comments.

Our detailed responses to comments are presented in the remainder of this letter. All of revisions have been highlighted in red in the revision.

1. Authors need to build the rational differentiating the variable response of microbial communities upon short term and long-term nitrogen addition – it wasn’t accordingly discussed.

Thank you for your advice. We have added the discussion about the variable response of microbial communities upon short-term and long-term nitrogen addition (lines 80-90 in the revision).

2. Key words should be precise and most relevant, for example site or region presence in key words may not be important.

We agree with your point of view that key words should be precise and most relevant. Follow your comments, we have deleted the site presence and added the soil microbial communities (line 33 in the revision).

3. Authors have mentioned about the response of microbes across forest and farmland as previously reported, assumption behind studying the short-term N addition in sandy grassland and semi-fixed sandy land ecosystems is to be mentioned.

Based on your comment, we have added the assumption in the revision (lines 95-98 in the revision).

4. The activity of β- 1,4-glucosidase was increased upon nitrogen addition (N5, N10), could this only be related to the release of nitrogen from plant litter degradation.

The activity of β- 1,4-glucosidase mainly releases glucose from cellulose and plays an important role in C cycling (lines 72-74 in the revision).The reason for the activity of β- 1,4-glucosidase was increased upon nitrogen addition (N5, N10) has shown in revision (lines 469-472 in the revision).

5. Why these two particular sites with distance of 1.5 km were chosen when the pedoclimatic condition relying on were (supposedly) the same.

The region’s sandy grassland grows on aeolian sandy soils or areas with sandy soils as the substrate and is typical of the grassland vegetation that develops in sandy land (Munkhdalai et al., 2007), the vegetation coverage is about 60%. The dominant plant species were annual herbs, including *Artemisia scoparia*, *Setaria viridis*, *Salsola collina*, and *Corispermum hyssopifolium*. Semi-fixed sandy land refers to the dune or sandy land where the vegetation coverage is between 10% and 29% and the distribution is uniform, and the movement of wind-sand flow is blocked, but the texture of quicksand still exists widely, the dominant plant species were perennial shrubs *Caragana microphylla* and annual herbs. Therefore, we tried to explore the response of soil microorganisms in different habitat types to N addition under the process of desertification restoration.

Munkhdalai, Z. A., Feng, Z. W., Wang, X. K., and Sun, H. W.: Sandy grassland blowouts in Hulunbuir, northeast China: geomorphology, distribution, and causes, Prog. Nat. Sci.-Mater., 17, 68–73, https://doi.org/10.1080/10020070612331343227, 2007.

6. For fungal amplification, 18S rRNA could be targeted?

Yes, 18S rRNA could be targeted for fungal amplification, meanwhile, 18S primers also detected the Vertebrata, Ciliophora, Arthropoda and Bicosoecida (Duan et al., 2018). In this study, we only focused on fungi amplification, therefore, we used ITS rRNA for fungal amplification in our research.

Duan, Y. L., Wu, F. S., Wang, W. F., Gu, J. D., Li, Y. F., Feng, H. Y., Chen, T., Liu, G. X., An, L. Z., 2018. Differences of microbial community on the wall paintings preserved in situ and ex situ of the Tiantishan Grottoes, China. Int. Biodeterior. Biodegrad.

7. Did authors check for inhibition test to remove the background noise before running the qPCR assays?

Yes, we have checked for inhibition test to make sure the accuracy of qPCR data (lines 204 in the revision).

8. The bacterial gene abundances quantified through 16S RNA gene copy numbers in the sandy grassland and semi-fixed sandy showed a high variation but were there no significant differences. The microbial community structure through targeting ITS region could be a possible way to know the differences in community structure across variable soils as discussed in taxonomic composition.

Thank you for your advice. This comment is great helpful to our future research. However, the bacterial gene abundances and the fungal gene abundances were quantified through 16S RNA and ITS rRNA, respectively. The result showed that the bacterial gene abundances and the fungal abundances in the sandy grassland and semi-fixed sandy were no significant differences (lines 507-508 in the revision). Therefore, ITS rRNA may not be as a possible way to explore the differences in community structure in present study.

9. Community composition analyses in nicely described but dominant phyla may be described in result section.

We have added the dominant phyla of the bacterial operational taxonomic units (OTUs) and fungal communities and highlighted in red (lines 342-345, 347, 352, 364-367 in the revision).

10. Figures could not be accessed as these were missing in the manuscript file so editor may verify the figures quality. I evaluated the work based on the detail of the manuscript provided.

We separately uploaded all the figures during the manuscript submission process, if these figures did not meet the requirements, we can re-upload all the figures to make them accessible.

11. The conclusions should not be repetition of results rather focus the key findings.

We have revised the description according to your comments (Line 542-553).

Thanks for your efforts to improve our manuscript. We hope that our replies and the resulting changes will be satisfactory, but we will be happy to work with you to resolve any remaining issues.

Sincerely,

Yulong Duan, Ph.D

Northwest Institute of Eco-Environment and Resources

Chinese Academy of Sciences

320 Donggang West Road, Lanzhou, 730000, China

Phone/Fax: 86-931-496-7219

E-mail: duanyulong@nieer.ac.cn
